# Associations Between Perceived Knowledge, Interprofessional Collaboration, Autonomy and the Frequency of Nursing Activities in Paediatric Extracorporeal Membrane Oxygenation: A Cross‐Sectional Study

**DOI:** 10.1111/nicc.70726

**Published:** 2026-10-16

**Authors:** Semyon Melnikov, Reem Abed Elhai, Nashaat Hijazi, Nagah Zaid, Alex Furmanov

**Affiliations:** ^1^ Department of Nursing Sciences, Steyer School of Health Professions Gray Faculty of Medical and Health Sciences, Tel Aviv University Tel Aviv Israel; ^2^ Nursing Management, Schneider Children's Medical Centre Petach Tikvah Israel; ^3^ PICU Rambam Health Care Campus Haifa Israel; ^4^ PICU Sheba Medical Centre Tel HaShomer Israel; ^5^ Nursing Management, Hadassah Hebrew University Medical Centre Jerusalem Israel

**Keywords:** autonomy, ECMO, interprofessional collaboration, knowledge, nursing, paediatric nursing

## Abstract

**Background:**

Extracorporeal membrane oxygenation (ECMO) requires coordinated multidisciplinary care. Although registered nurses (RNs) play a critical role, evidence on factors associated with the frequency of ECMO‐related nursing activities in paediatric settings remains limited.

**Aim:**

To examine associations between perceived ECMO knowledge, interprofessional collaboration, nursing autonomy and the frequency of ECMO‐related nursing activities.

**Study Design:**

A multicentre cross‐sectional study was conducted. RNs completed questionnaires assessing perceived ECMO knowledge, autonomy, interprofessional collaboration and ECMO‐related nursing activities. Data were analysed using bivariate correlations and multivariable linear regression models.

**Results:**

A convenience sample of 83 RNs from four Israeli paediatric intensive care units (median age 36 years (IQR 31–43), 84% female) participated in the study. In bivariate analyses, nursing autonomy was positively correlated with total ECMO nursing activities (Spearman's *r*_s_ = 0.34, *p* < 0.01), while interprofessional collaboration was also positively associated (*r*_s_ = 0.32, *p* < 0.01). In multivariable analyses, nursing autonomy was associated with greater involvement in administering medication and blood products through the ECMO circuit (*B* = 0.50, 95% CI [0.21, 0.80], *p* < 0.001), while perceived ECMO knowledge was associated with greater involvement in device calibration and monitoring (*B* = 0.39, 95% CI [0.13, 0.64], *p* = 0.004). RN–perfusionist teamwork was negatively associated with medication and blood administration through the ECMO circuit (*B* = −0.29, 95% CI [−0.50, −0.08], *p* = 0.008).

**Conclusions:**

The frequency of nursing activities in paediatric ECMO care is associated with both individual‐level factors (knowledge and autonomy) and interprofessional dynamics. Targeted education and training aimed at enhancing ECMO‐related knowledge, nursing autonomy and interprofessional collaboration may support RNs' participation in ECMO‐related nursing activities.

**Relevance to Clinical Practice:**

ECMO‐specific education, clear role delineation and effective interprofessional collaboration may enhance RNs' participation in paediatric ECMO care.

## Impact Statements

- What is known about the topic
  ○ RNs play a central role in paediatric ECMO care, but their involvement varies across ECMO‐related activities.
  ○ Knowledge, autonomy and interprofessional collaboration are thought to influence nursing participation, although evidence in paediatric ECMO is limited.
- What this paper adds
  ○ Nursing autonomy and perceived ECMO knowledge are key‐associated factors of nurses' involvement in ECMO‐related activities.
  ○ RNs are more involved in routine ECMO monitoring than in medication/blood administration or emergency ECMO tasks.
  ○ RN–perfusionist teamwork may limit nurses' involvement in certain shared ECMO responsibilities, highlighting complex interprofessional dynamics.

## 1 Introduction

Extracorporeal membrane oxygenation (ECMO) is a life‐saving intervention used in patients with reversible, refractory cardiopulmonary failure [1]. ECMO provides extracorporeal gas exchange by removing deoxygenated blood, oxygenating it and returning it to the venous or arterial circulation. According to the ELSO (Extracorporeal Life Support Organization) Registry (2009–2022), 70.0% of ECMO runs were performed in adults, 16.7% in paediatric patients and 13.3% in neonates [2]. In paediatric populations, the main indications for ECMO include pneumonia, acute respiratory distress syndrome and congenital heart disease [3, 4].

Successful ECMO care in the paediatric intensive care unit (PICU) requires coordinated multidisciplinary teamwork involving physicians, respiratory therapists, perfusionists and nurses [5]. ECMO specialist nurses play a central role in the preparation, initiation and ongoing management of ECMO therapy [6]. Their responsibilities include assisting with ECMO initiation, monitoring the circuit, responding to emergencies and ensuring patient safety. Variability in these activities may affect patient safety, timely intervention and ECMO effectiveness; for example, less frequent monitoring may delay detection of circuit, bleeding or neurological complications [7].

### 1.1 Background

Although a substantial body of research has examined ECMO outcomes, complications and clinical management in critical care settings [8, 9], far fewer studies have specifically examined nurses' roles, workload and bedside activities in ECMO care [10, 11, 12]. Existing quantitative studies have primarily focused on nursing workload rather than the frequency of specific ECMO nursing activities. Previous studies have shown that nursing workload during ECMO is influenced by factors such as COVID‐19, prone positioning, delayed ECMO initiation and continuous renal replacement therapy [7, 13]. However, these studies focused on overall workload rather than the frequency of specific ECMO nursing activities or the individual and organisational factors. Studies in adult ECMO settings have shown that perceived ECMO knowledge has been positively associated with the frequency of nursing activities across multiple domains [14], while education and continuous training have been shown to enhance RNs' engagement in ECMO management [7]. In addition, nursing autonomy has been linked to greater participation in complex clinical decision‐making [15, 16] and interprofessional collaboration has been identified as a key factor influencing communication, coordination and overall care delivery in ECMO settings [17, 18].

However, it remains unknown whether these associations apply to paediatric ECMO care, where organisational structures, team composition and nursing roles may differ substantially from adult settings.

### 1.2 Aims and Objectives

The aim of this study was to examine factors associated with the frequency of RNs' activities in caring for paediatric patients supported by ECMO. The objectives were to (1) describe the frequency of RNs' ECMO‐related nursing activities; and (2) examine associations between perceived ECMO knowledge, interprofessional collaboration, nursing autonomy and the frequency of ECMO‐related nursing activities.

## 2 Design and Methods

This cross‐sectional study was conducted in accordance with the STROBE guidelines [19].

### 2.1 Setting and Sample

A convenience sample of RNs working in paediatric intensive care units (PICUs) was recruited from four tertiary medical centres in Israel: Hadassah, Rambam, Schneider and Sheba. The inclusion criteria were RNs working in one of the participating PICUs who had completed standardised ECMO training and practised in the ECMO specialist nurse role. RNs who had not completed ECMO training or did not practise in this role were excluded. In this study, the term ‘registered nurses (RNs)’ refers to registered nurses who provide care for ECMO‐supported patients and have completed standardised ECMO training. An a priori sample size calculation using G*Power [20] indicated that 85 participants were required to achieve 80% power for multiple linear regression with four independent variables, assuming a medium effect size (*f* ^2^ = 0.15) and *α* = 0.05.

Eligibility criteria included completion of an accredited ECMO course approved by the Israeli Ministry of Health, consisting of 24 h of theoretical instruction and 16 h of clinical training. In Israel, RNs who complete this accredited ECMO training are formally qualified to practise in the ECMO specialist nurse role. Accordingly, all participants in this study were RNs practising in the ECMO specialist nurse role. Data were collected between January 2024 and June 2025. Data were collected over an extended period to allow recruitment of a sufficient number of ECMO‐trained RNs; no major changes in ECMO protocols or training procedures were reported across centres during this time. A pilot study with five RNs confirmed questionnaire clarity, and no revisions were required.

A total of 100 questionnaires were distributed across centres, with approximately 25 per centre; slightly more questionnaires were distributed at Schneider due to higher availability of eligible RNs. Participation was voluntary, informed consent was obtained and confidentiality was assured. Although participants were recruited from multiple centres, centre‐level analyses were not performed due to the relatively small sample size within each centre; therefore, potential centre effects could not be examined. To reduce potential selection bias, participants were recruited from four tertiary ECMO centres. To minimise response bias, participation was voluntary, and no directly identifying information was collected.

### 2.2 Data Collection Tools and Methods

Data were collected by a structured self‐administered questionnaire comprising six sections: (1) nursing activities in the care of ECMO‐supported patients, (2) interprofessional collaboration between RNs and physicians and other RNs in ECMO care scale, (3) perceived ECMO knowledge, (4) perceived RN‐perfusionist cooperation, (5) nursing autonomy and (6) the respondent's demographic data. Sections 1 and 5 were based on validated tools taken from the published literature. Sections 2, 4 were developed by the authors based on a literature review and relevant themes. Given the absence of validated instruments specific to paediatric ECMO care, some scales were developed or adapted for this study. Therefore, exploratory factor analysis was performed to obtain preliminary evidence regarding their underlying factor structure before their use in the main analyses. These psychometric analyses were conducted using the same study sample and were intended to support the use of the instruments in this study rather than to provide formal instrument validation. Higher scores reflected higher levels of the respective constructs, and no established cut‐off points are available for these scales.

#### 2.2.1 Nursing Activities in the Care of ECMO‐Supported Patients Scale

This 20‐item scale assesses the frequency of nursing activities related to ECMO care across four domains: ECMO device calibration and monitoring, medication and blood administration through the ECMO circuit, emergency ECMO activities and general nursing care not directly related to the ECMO device [15]. Responses are rated on a 5‐point Likert scale (1 = never to 5 = always). Subscale and total scores are calculated as the mean of the corresponding items (range 1–5), with higher scores indicating greater frequency of ECMO‐related nursing activities. The scale has been previously described and validated in Israeli intensive care RNs [15]. The previously validated 20‐item version was used without modification in the present study. In the present study, Cronbach's α ranged from 0.62 to 0.92 across the subscales and was 0.82 for the total scale.

#### 2.2.2 Interprofessional Collaboration Between RNs and Physicians and Other RNs in ECMO Care Scale

This five‐item scale was developed for the present study to assess perceived collaboration, trust and informational support received by RNs from multidisciplinary team members while caring for paediatric patients supported by ECMO in the PICU. Responses were rated on a 5‐point Likert scale (1 = strongly disagree to 5 = strongly agree). A total score was calculated as the mean of all items (range 1–5), with higher scores indicating greater perceived collaboration and support. Exploratory factor analysis supported a two‐factor structure, comprising ICU team collaboration and support and communication and decision‐making with physicians. Internal consistency was acceptable (overall Cronbach's *α* = 0.77). The full scale is provided in Appendix S1a. Given the relatively small sample size, exploratory factor analyses conducted in this study should be considered preliminary and interpreted with caution.

#### 2.2.3 Perceived Knowledge, Confidence and Skill in ECMO Care Scale

This scale was adapted from the instrument developed by Breeding et al. [21] The original instrument comprised five items assessing perceived knowledge of VV and VA ECMO, confidence in caring for ECMO patients and managing ECMO emergencies, and skill in managing ECMO circuit problems, rated on a 9‐point scale. For the present study, the confidence and skill domains were expanded into more specific ECMO care situations relevant to clinical practice, resulting in a 10‐item scale, and the response format was changed to a 5‐point scale (1 = worst possible to 5 = best possible). A total score is calculated as the mean of all items (range 1–5), with higher scores indicating greater perceived ECMO knowledge, confidence and skills. Exploratory factor analysis supported a one‐factor structure, and internal consistency was excellent (Cronbach's *α* = 0.96). The full scale is provided in Appendix S1b.

#### 2.2.4 RNs—Perfusionists Teamwork Scale

The scale was developed for the present study because no suitable validated instrument specifically assessing teamwork between RNs and perfusionists in ECMO care was identified. The items were initially developed by three authors and subsequently reviewed and approved by all authors for relevance and clarity in the context of paediatric ECMO care. This six‐item scale assesses perceived teamwork, availability, communication and role clarity between RNs and ECMO perfusionists during paediatric ECMO care. Items are rated on a 4‐point Likert scale (1 = strongly disagree to 4 = strongly agree). A total score is calculated as the mean of all items (range 1–4), with higher scores indicating better perceived teamwork. Exploratory factor analysis supported a one‐factor structure, and internal consistency was excellent (Cronbach's *α* = 0.96). The full scale is provided in Appendix S1c.

#### 2.2.5 Nursing Work Index‐Revised (NWI‐R), Autonomy Subscale

The Autonomy subscale of the NWI‐R [22] consists of five items assessing RNs' autonomy. Items are rated on a 4‐point Likert scale (1 = strongly disagree to 4 = strongly agree). A total score is calculated as the mean of all items (range 1–4), with higher scores indicating greater perceived nursing autonomy. Internal consistency of the autonomy subscale was previously reported as Cronbach's *α* = 0.75 [22]; in the present study, Cronbach's α was 0.96.

#### 2.2.6 Demographic and Professional Characteristics

This section included items on participants' age, gender, years of clinical experience, years of PICU experience and ECMO‐related training and experience.

All scale scores were subsequently standardised (see Data Analysis section) to enable comparability across measures with different response ranges. There were no missing data for the study variables, as all returned questionnaires were complete.

### 2.3 Data Analysis

To enable comparability across measures with different Likert‐scale ranges (e.g., 1–4 and 1–5), all scale scores were standardised to z‐scores prior to analysis using the formula: *Z* = (*X* − *μ*)/*σ*, where *X* represents the individual raw score, *μ* the scale mean and *σ* the scale standard deviation. For ease of interpretation and graphical presentation, z‐scores were subsequently rescaled to a 0–100 range using the transformation: 50 + (*Z* × 10).

All multi‐item Likert‐scale variables were treated as continuous measures, consistent with the widely accepted use of parametric methods for composite Likert‐scale scores [23]. Variable distributions were assessed using the Shapiro–Wilk test and visual inspection of residual plots (including Q–Q plots). For group comparisons involving non‐normally distributed continuous variables, Kruskal–Wallis tests were used. When significant, post hoc pairwise comparisons were performed using Wilcoxon rank‐sum tests with Holm adjustment for multiple comparisons. Accordingly, Spearman's rank correlation coefficients (rs) were used to examine associations among the study variables. Sociodemographic variables were analysed separately using univariate linear regression models. Univariate linear regressions assessed associations between independent variables and each ECMO activity domain. All demographic, professional, organisational and psychosocial variables measured in the study were initially examined in univariate linear regression analyses for their association with each ECMO nursing activity outcome. Variables with *p* < 0.10 in univariable analyses were considered for inclusion in multivariable models to reduce the risk of excluding potentially relevant associated factors at an early stage, following the general principle of liberal initial screening in exploratory model building [24]. The multivariable analyses were conducted to examine the independent associations between the study variables and the frequency of ECMO‐related nursing activities. This approach allowed potential confounding by other measured variables retained in the multivariable models to be taken into account. These analyses were exploratory in nature and were not intended to develop predictive models or support causal inference. Multivariable linear regression models were then constructed using a stepwise selection procedure (both forward and backward) based on Akaike's information criterion (AIC). Model assumptions were assessed, including linearity, normality of residuals and homoscedasticity. Potential overlap between collaboration‐related variables was examined, and multicollinearity diagnostics indicated acceptable levels. Residual diagnostics (Shapiro–Wilk tests) indicated acceptable normality with only minor deviations. All tests were two‐tailed with significance set at *p* < 0.05. Analyses were conducted using R software (version 4.4.1).

### 2.4 Ethics Statement

Ethical approval was obtained from the Helsinki Committees of four medical centresu—Hadassah Medical Centre, Jerusalem (HMO‐0075‐23; approved on July 11, 2024), Rambam Health Care Campus, Haifa (RMB‐0074‐24; approved on June 3, 2025), Schneider Childrenu's Medical Centre, Petach Tikva (#RMC‐0902‐23; approved on January 29, 2024), and Sheba Medical Centre, Ramat Gan (#SMC‐0389‐23; October 21, 2024)—all approvals were provided prior to study initiation.

## 3 Results

### 3.1 Demographic and Professional Characteristics of the Participants

Table 1 presents the participants' demographic and professional characteristics. The participants' median age was 36 years (IQR 31–43); 84% were women and 73% were employed at 75%–90% or full time (100%). Sixty‐four per cent of the RN participants held a BA or BSN degree; all had completed an ECMO course, the majority (98%) had completed an intensive care course and 70% had taken an ECMO refresher course in the past year. Participants had a median seniority of 11 years in nursing (IQR 6–18), 8 years in intensive care (IQR 5–16) and 5 years working with ECMO (IQR 2–10). The final sample size (*n* = 83) was slightly below the target (*n* = 85). The overall response rate was 83%, with participation from Hadassah (*n* = 10), Rambam (*n* = 25), Schneider (*n* = 27) and Sheba (*n* = 21).

**TABLE 1:** Sociodemographic characteristics (*N* = 83).

	*n*	%
Gender		
Female	70	84
Male	13	16
Country of birth		
Israel	65	78
Other	18	22
Partner		
Yes	61	73
No	19	23
Missing	3	4
Education		
RN	1	1
RN BA/BSN	53	64
RN MA/MSN/PhD	29	35
Intensive care course		
Yes	81	98
No	2	2
ECMO course		
Yes	83	100
ECMO refresh course in the last year		
Yes	58	70
No	24	29
Missing	1	1
Managerial position (head nurse/deputy)		
Yes	8	10
No	75	90
Job time (%)		
100	27	33
75–90	33	40
66	13	16
50	9	11
Other	1	1
		**Median [IQR]**
Age (years)		36 [31, 43]
Seniority (years)		11 [6,18]
Seniority in the intensive care (years)		8 [5,16]
Seniority working with ECMO (years)		5 [2,10]

Abbreviation: IQR, interquartile range.

### 3.2 Associations Between the Study Variables

Table 2 presents descriptive statistics and correlations among the study variables, while Figure 1 illustrates the distribution of ECMO activity domain scores and the results of pairwise comparisons between domains. Median engagement was highest for nursing care of ECMO‐supported patients not related to the device (median = 100, IQR = 84.52–100), followed by ECMO device calibration and monitoring (median = 62.5, IQR = 50.00–68.75). Engagement was substantially lower for emergency ECMO activities (median = 30.0, IQR = 15.94–45.00) and medication/blood administration through the ECMO circuit (median = 31.25, IQR = 12.50–48.44). A Kruskal–Wallis test demonstrated significant differences across the four ECMO activity domains (*χ* ^2^ [3] = 162.06, *p* < 0.001), with all pairwise comparisons significant except between emergency ECMO activities and medication/blood administration (Figure 1).

**TABLE 2:** Spearman correlation coefficients (*r*_s_) among study variables (*N* = 83).

Variable	Md [IQR]	Observed range	ECMO calibration/monitoring	ECMO medication/blood administration	ECMO in emergency	Nursing care not related to the ECMO	Total ECMO nursing activities	Nursing team support	MD support/decision‐making	Interprofessional collaboration and support total	RN‐perfusionist teamwork	Perceived ECMO knowledge	Autonomy
ECMO calibration/monitoring	62.50 [50.00, 68.75]	6.25–100.00	—										
ECMO medication/ blood administration	31.25 [12.50, 48.44]	0.00–100.00	0.50***	—									
ECMO in emergency	30.00 [15.94, 45.00]	0.00–100.00	0.31**	0.28*	—								
Nursing care not related to the ECMO	100.00 [84.52, 100.00]	42.86–100.00	0.23*	0.30**	0.23	—							
Total ECMO nursing activities	61.25 [52.50, 67.81]	26.25–100.00	0.62***	0.68***	0.75***	0.48***	—						
Nursing Team Support	100.00 [77.08, 100.00]	25.00–100.00	0.31**	0.19	0.21	0.37***	0.29**	—					
MD support/decision‐making	75.00 [75.00, 100.00]	50.00–100.00	0.17	0.08	0.17	0.36***	0.24*	0.48***	—				
Interprofessional Collaboration and Support total	90.00 [75.00, 100.00]	50.00–100.00	0.28**	0.14	0.26*	0.42***	0.32**	0.87***	0.83***	—			
RN–Perfusionist teamwork	77.78 [61.11, 94.44]	0.00–100.00	0.09	−0.21	0.11	0.23*	0.09	0.17	0.28*	0.27*	—		
Perceived ECMO knowledge	75.00 [58.75, 80.42]	22.50–100.00	0.33**	0.06	0.24*	0.07	0.21	0.08	0.17	0.14	0.29**	—	
Autonomy	73.33 [60.00, 80.00]	33.33–100.00	0.20	0.33**	0.22*	0.31**	0.34**	0.54***	0.39***	0.55***	0.19	0.06	—

*Note:* Values represent Spearman's rank correlation coefficients (*r*_s_). Md—median, IQR—interquartile range, *p < 0.05, **p < 0.01, ***p < 0.001. Bold values indicate statistically significant correlations (*p* < 0.05).

**FIGURE 1:**
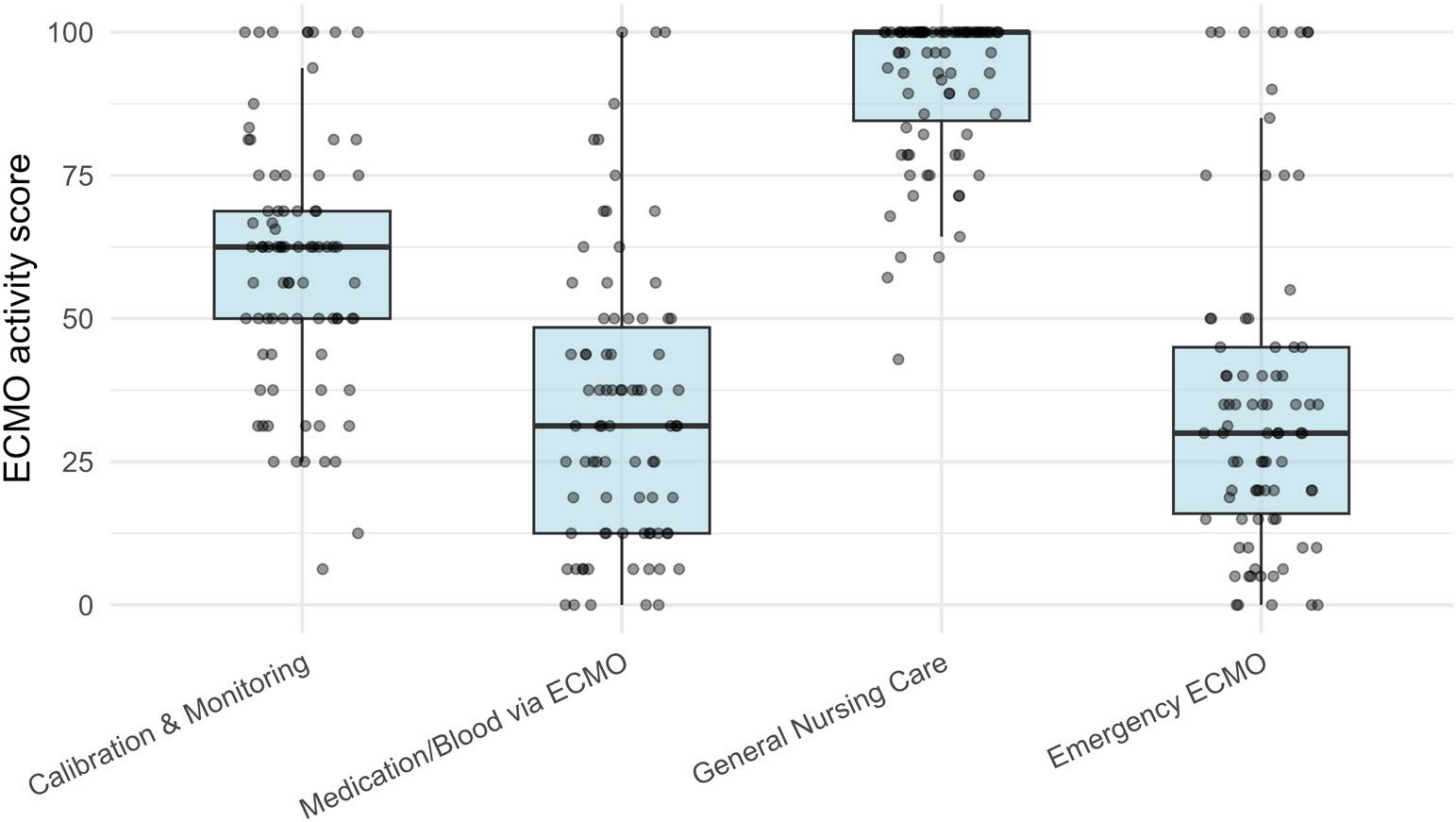
Distribution of ECMO nursing activity domain scores. Boxplots represent the median and interquartile range of standardised ECMO activity scores (0–100), with higher scores indicating a higher frequency of nursing activities in each domain. Pairwise comparisons between domains were conducted following a significant Kruskal–Wallis test.

ECMO calibration and monitoring was positively associated with interprofessional collaboration (*r*_s_ = 0.28, *p* < 0.01), particularly nursing team support (*r*_s_ = 0.31, *p* < 0.01), and perceived ECMO knowledge (*r*_s_ = 0.33, *p* < 0.01) (Table 2). Medication and blood administration through ECMO was positively associated with nursing autonomy (*r*_s_ = 0.33, *p* < 0.01). Emergency ECMO activities were positively associated with interprofessional collaboration (*r*_s_ = 0.26, *p* < 0.05), perceived ECMO knowledge (*r*_s_ = 0.24, *p* < 0.05) and nursing autonomy (*r*_s_ = 0.22, *p* < 0.05). Nursing care not related to the ECMO device showed the strongest associations with interprofessional collaboration overall (*r*_s_ = 0.42, *p* < 0.001). This included nursing team support (*r*_s_ = 0.37, *p* < 0.001), physician support (*r*_s_ = 0.36, *p* < 0.001), RN–perfusionist teamwork (*r*_s_ = 0.23, *p* < 0.05) and nursing autonomy (*r*_s_ = 0.31, *p* < 0.01). Total ECMO nursing activities were positively correlated with interprofessional collaboration overall (*r*_s_ = 0.32, *p* < 0.01). This included nursing team support (*r*_s_ = 0.29, *p* < 0.01), physician support (*r*_s_ = 0.24, *p* < 0.05) and nursing autonomy (*r*_s_ = 0.34, *p* < 0.01).

Several factors were associated with multiple ECMO nursing activity domains. Nursing autonomy showed the most consistent associations across domains, while perceived ECMO knowledge and nursing team support were associated with selected activity domains. Overall, these findings suggest that both individual and team‐related factors are associated with the frequency of ECMO‐related nursing activities.

### 3.3 Univariate Analyses

Table 3 presents the results of univariate linear regression models examining associations between demographic/professional characteristics and the frequency of ECMO‐related nursing activities across four domains and total ECMO nursing activity scores. Several independent variables, including education, nursing autonomy, nursing team support and perceived ECMO knowledge, showed significant associations with one or more activity domains in the univariate analyses.

**TABLE 3:** Univariate regression results by dependent variable (*n* = 83).

Independent variables	ECMO calibration/monitoring	ECMO medication/blood admin.	ECMO in emergency	Not ECMO activities	Total ECMO nursing activities
Age (years)	0.08 [−0.56, 0.73], *p* = 0.795	−0.58 [−1.30, 0.15], *p* = 0.117	0.43 [−0.41, 1.28], *p* = 0.312	−0.25 [−0.62, 0.12], *p* = 0.190	−0.14 [−0.56, 0.28], *p* = 0.511
Gender (male/female)	−5.50 [−18.76, 7.75], *p* = 0.411	−13.22 [−28.01, 1.57], *p* = 0.079	−1.50 [−18.91, 15.92], *p* = 0.865	6.06 [−1.58, 13.70], *p* = 0.118	−1.36 [−10.09, 7.38], *p* = 0.758
Partner (yes/no)	0.22 [−11.40, 11.84], *p* = 0.970	−4.60 [−17.87, 8.67], *p* = 0.492	−10.26 [−25.49, 4.97], *p* = 0.184	−2.49 [−9.32, 4.33], *p* = 0.469	−5.16 [−12.79, 2.47], *p* = 0.182
Country of birth (Israel/other)	8.61 [−2.98, 20.19], *p* = 0.143	4.84 [−8.42, 18.10], *p* = 0.470	4.21 [−11.13, 19.56], *p* = 0.586	1.37 [−5.46, 8.20], *p* = 0.691	3.69 [−3.98, 11.36], *p* = 0.341
Education	−46.08 [−89.53, −2.64], ***p* = 0.038**	−74.14 [−121.58, −26.70], ***p* = 0.003**	7.97 [−50.94, 66.89], *p* = 0.788	−11.82 [−37.65, 14.01], *p* = 0.365	−26.01 [−54.75, 2.73], *p* = 0.075
ECMO seniority (years)	0.70 [−0.30, 1.70], *p* = 0.165	−0.25 [−1.39, 0.90], *p* = 0.671	0.56 [−0.77, 1.89], *p* = 0.403	−0.08 [−0.65, 0.49], *p* = 0.779	0.08 [−0.57, 0.73], *p* = 0.810
ICU seniority (years)	0.26 [−0.44, 0.95], *p* = 0.462	−0.37 [−1.15, 0.42], *p* = 0.354	0.67 [−0.23, 1.57], *p* = 0.140	−0.01 [−0.41, 0.40], *p* = 0.974	0.06 [−0.39, 0.52], *p* = 0.777
Seniority (years)	0.18 [−0.42, 0.79], *p* = 0.552	−0.48 [−1.16, 0.20], *p* = 0.164	0.54 [−0.24, 1.33], *p* = 0.173	−0.14 [−0.49, 0.21], *p* = 0.420	−0.05 [−0.45, 0.35], *p* = 0.803
Nursing autonomy	0.35 [0.07, 0.63], ***p* = 0.013**	0.48 [0.17, 0.78], ***p* = 0.003**	0.24 [−0.13, 0.61], *p* = 0.206	0.25 [0.09, 0.40], ***p* = 0.003**	0.30 [0.14, 0.47], ***p* = 0.001**
Nursing Team Support	0.43 [0.11, 0.75], ***p* = 0.008**	0.23 [−0.15, 0.61], *p* = 0.227	0.42 [−0.01, 0.86], *p* = 0.056	0.27 [0.08, 0.46], ***p* = 0.006**	0.31 [0.10, 0.53], ***p* = 0.005**
MDs support	0.23 [−0.05, 0.52], *p* = 0.102	0.02 [−0.31, 0.35], *p* = 0.905	0.14 [−0.24, 0.52], *p* = 0.467	0.21 [0.04, 0.38], ***p* = 0.014**	0.16 [−0.03, 0.35], *p* = 0.105
Collaboration total	0.49 [0.14, 0.85], ***p* = 0.008**	0.19 [−0.24, 0.63], *p* = 0.376	0.43 [−0.07, 0.92], *p* = 0.092	0.36 [0.14, 0.57], ***p* = 0.001**	0.35 [0.11, 0.60], ***p* = 0.005**
Perceived ECMO knowledge	0.45 [0.19, 0.70], ***p* = 0.001**	0.22 [−0.10, 0.54], *p* = 0.174	0.37 [0.01, 0.74], ***p* = 0.042**	0.12 [−0.05, 0.28], *p* = 0.164	0.22 [0.04, 0.40], ***p* = 0.018**
RN–Perfusionist teamwork	0.09 [−0.11, 0.29], *p* = 0.387	−0.26 [−0.48, −0.04], ***p* = 0.023**	0.20 [−0.06, 0.46], *p* = 0.126	0.09 [−0.03, 0.21], *p* = 0.137	0.05 [−0.08, 0.18], *p* = 0.426

*Note:* Coefficients represent unstandardised *B* (95% CI) from linear regression. For categorical independent variables, coefficients represent mean differences compared with the reference group (reference = male for gender; MSc + for education). *B* = unstandardised regression coefficient. Bold values indicate statistical significance (*p* < 0.05).

### 3.4 Multivariable Analyses

Table 4 presents the multivariable regression results including associated factors with *p* < 0.10 in univariate analyses. In the multivariable analyses, nursing autonomy remained positively associated with medication/blood administration through ECMO and total ECMO nursing activities. Perceived ECMO knowledge was positively associated with ECMO calibration/monitoring and total activities, whereas RN–perfusionist teamwork was negatively associated with medication/blood administration through ECMO. These findings indicate that RNs' involvement in ECMO care is associated with autonomy and perceived knowledge, while interprofessional dynamics with perfusionists may be related to lower engagement in certain procedures. Other variables that were significant in the univariate analyses did not remain statistically significant in the multivariable models.

**TABLE 4:** Multivariate regression results by dependent variable (*n* = 83).

Independent variables	ECMO calibration/monitoring	ECMO medication/blood admin.	ECMO in emergency	Not ECMO activities	Total ECMO nursing activities
Gender (male/female)		−11.49 [−24.72, 1.73], *p* = 0.092			
Education	−7.28 [−16.03, 1.47], *p* = 0.107	−7.1 [−17.31, 3.1], *p* = 0.177			−2.7 [−8.41, 3.0], *p* = 0.356
Nursing autonomy	0.08 [−0.22, 0.38], *p* = 0.604	0.5 [0.21, 0.8], ***p* = 0.001**		0.17 [−0.02, 0.36], *p* = 0.087	0.2 [0.0, 0.4], ***p* = 0.050**
Nursing team support	0.25 [−0.35, 0.86], *p* = 0.413		0.46 [−0.4, 1.33], *p* = 0.298	−1264.44 [−2903.38, 374.51], *p* = 0.135	0.18 [−0.22, 0.57], *p* = 0.375
MDs support				−842.98 [−1935.67, 249.71], *p* = 0.135	
Collaboration total	0.01 [−0.71, 0.72], *p* = 0.984		−0.13 [−1.13, 0.87], *p* = 0.798	2107.62 [−623.99, 4839.24], *p* = 0.135	−0.04 [−0.5, 0.43], *p* = 0.879
Perceived ECMO knowledge	0.39 [0.13, 0.64], ***p* = 0.004**		0.34 [−0.02, 0.7], *p* = 0.071		0.21 [0.04, 0.37], ***p* = 0.016**
RN–Perfusionist teamwork		−0.29 [−0.5, −0.08], ***p* = 0.008**			

*Note:* Values are unstandardised coefficients (*B*) with 95% confidence intervals (CI) and *p*‐values. Each column represents a dependent variable, and each row represents an independent variable. Bold values indicate statistical significance (*p* < 0.05).

## 4 Discussion

This study examined associations between perceived ECMO knowledge, interprofessional collaboration, nursing autonomy and the frequency of RNs' activities in the care of paediatric patients supported by ECMO. Overall, RNs reported greater engagement in general PICU care than in ECMO‐specific activities, while perceived ECMO knowledge and nursing autonomy emerged as the factors most consistently associated with ECMO‐related nursing activities.

RNs reported significantly higher participation in ECMO calibration and monitoring compared with medication/blood administration and emergency ECMO activities. These responsibilities align closely with RNs' traditional monitoring role in the ICU, which likely explains the higher frequency of involvement. In contrast, medication and blood administration through the ECMO circuit may be performed less frequently by RNs because these activities often require additional clinical oversight. Similarly, emergency ECMO activities occur unpredictably, demand advanced expertise and are not part of routine care, which may limit RNs' exposure and confidence in these tasks [25].

Perceived ECMO knowledge was positively associated with RNs' involvement in ECMO calibration and monitoring and total ECMO‐related activities. This finding is consistent with previous adult ECMO studies showing that greater perceived knowledge and ongoing education are associated with more active participation in ECMO care [14, 26]. Qualitative studies further suggest that knowledgeable nurses are more likely to undertake technical ECMO procedures rather than defer them to physicians or perfusionists [11].

Compared with adult ECMO settings, paediatric ECMO care involves distinct clinical, organisational and family‐related factors that may influence nursing roles and interprofessional collaboration [27, 28]. Therefore, findings derived from adult ECMO settings should be applied cautiously to paediatric populations, and further research is needed to better understand these context‐specific factors.

Nursing autonomy was positively associated with RNs' involvement in medication and blood administration through the ECMO circuit and with total ECMO activities. Previous research has shown that greater autonomy facilitates management of complex clinical situations [15, 29]. Greater autonomy may facilitate participation in medication and blood administration through the ECMO circuit by increasing confidence and accountability.

RN–perfusionist teamwork was negatively associated with nurses' involvement in medication and blood administration through ECMO. This finding may reflect task specialisation, whereby perfusionists assume primary responsibility for ECMO circuit‐related procedures, including medication and blood administration, as part of clearly defined professional roles.

Several variables, including nursing team support, physician support and overall collaboration, were associated with ECMO activities in univariate analyses but did not remain significant in multivariable models, while RN–perfusionist teamwork remained associated with medication and blood administration only in the multivariable model. These patterns may reflect overlap and complex relationships among the study variables. Given the cross‐sectional design and potential influence of centre‐specific organisational practices, no conclusions can be drawn regarding causal or mediating relationships.

### 4.1 Strengths and Limitations

Strengths include the multivariable analysis and the focus on paediatric ECMO nursing, an understudied area.

Limitations include the modest sample size, which may have reduced power to detect smaller effects, and the cross‐sectional design, which precludes causal inference. All key variables were self‐reported and collected at a single time point, which may have introduced social desirability and common method bias, potentially inflating the observed associations. Furthermore, the frequency of ECMO‐related nursing activities should not be interpreted solely as a reflection of individual nurse engagement. It may also be influenced by patient acuity, institutional protocols, staffing levels and task allocation within the multidisciplinary ECMO team. The use of a convenience sample may limit the representativeness of the findings, although participants were recruited from four tertiary ECMO centres and all had completed standardised ECMO training. Several instruments were newly developed or adapted and evaluated within the same sample, which may increase the risk of overfitting and limit the generalisability of the psychometric findings. The ECMO device calibration and monitoring subscale showed modest internal consistency (Cronbach's α = 0.62); therefore, related findings should be interpreted with caution. Stepwise regression with a relatively small sample may have increased the risk of overfitting; therefore, the multivariable findings should be interpreted as exploratory. Additionally, findings may not be generalizable beyond paediatric ECMO or the specific organisational and cultural context of Israeli PICUs.

### 4.2 Future Research and Implications

Future studies should include larger multicentre samples and longitudinal or interventional designs to clarify the relationships between knowledge, autonomy, interprofessional collaboration and ECMO‐related nursing activities. Qualitative research may further explore interprofessional collaboration within ECMO teams, particularly between RNs and perfusionists.

### 4.3 Relevance to Clinical Practice

These findings highlight the potential value of strengthening ECMO‐specific education, promoting nursing autonomy and supporting effective interprofessional collaboration to optimise ECMO‐related nursing activities. Clear role delineation between RNs and perfusionists may also help define responsibilities for shared ECMO‐related activities.

## 5 Conclusion

This study suggests that nursing autonomy and perceived ECMO knowledge are important factors associated with the frequency of ECMO‐related nursing activities among RNs caring for paediatric patients supported by ECMO. At the same time, the negative association between RN–perfusionist teamwork and selected ECMO activities highlights the complexity of interprofessional dynamics within ECMO teams. These findings support further investigation of education, role delineation and collaborative models to optimise ECMO‐related nursing activities.

## Funding

The authors have nothing to report.

## Consent

Informed consent was obtained from all participants prior to data collection. Permission to Reproduce Material From Other Sources: Not applicable. No copyrighted material from other sources was reproduced in this manuscript. Clinical Trial Registration: Not applicable. This study was observational and did not involve a clinical trial.

## Conflicts of Interest

The authors declare no conflicts of interest.

## Supporting information


**Appendix S1:** (a) Interprofessional collaboration between nurses and physicians and other nurses in ECMO Care Scale. (b) Perceived Knowledge, Confidence and Skill in ECMO Care Scale. (c) Nurses—Perfusionists Teamwork Scale Perceived collaboration between nurses and perfusionists in the care of patients supported by ECMO.

## Data Availability

The data that support the findings of this study are available from the corresponding author upon reasonable request.
