# Supplementary material for: Associations Between Perceived Knowledge, Interprofessional Collaboration, Autonomy and the Frequency of Nursing Activities in Paediatric Extracorporeal Membrane Oxygenation: A Cross‐Sectional Study
Source: Nurs Crit Care. 2026 Oct 16;31(6):e70726. doi: 10.1111/nicc.70726 (PMC13673569; doi:10.1111/nicc.70726)
Supplement: Supplementary file 1 [file NICC-31-0-s001.docx]

**Supplementary Appendix I**

a. **Interprofessional collaboration between nurses and physicians and other nurses in ECMO Care Scale.**

Below are statements related to your work in the paediatric intensive care unit (PICU) with patients supported by ECMO. Please rate your level of agreement on a scale from 1 (strongly disagree) to 5 (strongly agree).

1. The PICU team greatly assists me in providing care to patients supported by ECMO.
2. The PICU team greatly assists me in operating the ECMO device
3. When caring for a patient supported by ECMO in the intensive care unit, I rely heavily on experienced nurses
4. When caring for a patient supported by ECMO in the intensive care unit, I rely heavily on physicians
5. Physicians in the unit provide me with important information and advice related to working with ECMO
b. **Perceived Knowledge, Confidence and Skill in ECMO Care Scale.**

Please select the response that best reflects your perception of each statement on a scale from 1 (the worst) to 5 (the best).

1. Your level of knowledge regarding VV ECMO
2. Your level of knowledge regarding VA ECMO
3. Your level of confidence in caring for a patient supported by VV ECMO
4. Your level of confidence in caring for a patient with percutaneous VA ECMO
5. Your level of confidence in caring for a patient with surgically implanted VA ECMO
6. Your level of confidence in admitting and caring for a newly admitted patient supported by ECMO
7. Your level of confidence in caring for a hemodynamically unstable patient supported by ECMO
8. Your level of confidence in caring for a patient supported by ECMO with a jugular cannula
9. Your level of confidence in managing emergency situations in a patient supported by ECMO
10. Your level of confidence in troubleshooting ECMO system problems
c. **Nurses - Perfusionists Teamwork Scale**

**Perceived collaboration between nurses and perfusionists in the care of patients supported by ECMO**

Please indicate your level of agreement regarding the occurrence of this phenomenon in the PICU where you cared for a child supported by ECMO. Responses should be rated on a scale from 1 (strongly disagree) to 4 (strongly agree).

1. There is collaboration between nurses and the perfusionist, and patients are cared for as one team (team-based care)
2. The perfusionist is available when needed
3. The perfusionist’s response time when called is adequate for providing timely and high-quality care to a child supported by ECMO
4. There are good working relationships between nurses and perfusionists
5. The presence and collaboration of the perfusionist provide a sense of safety in patient care
6. The authority and responsibilities of perfusionists are clear and well defined to both the nursing staff and the perfusion team
